# Understanding the Frying Process of Plant-Based Foods Pretreated with Pulsed Electric Fields Using Frying Models

**DOI:** 10.3390/foods9070949

**Published:** 2020-07-17

**Authors:** Zihan Xu, Sze Ying Leong, Mohammed Farid, Patrick Silcock, Phil Bremer, Indrawati Oey

**Affiliations:** 1 Department of Food Science, University of Otago, PO Box 56, 9054 Dunedin, New Zealand; xuzhfood@163.com (Z.X.); sze.leong@otago.ac.nz (S.Y.L.); pat.silcock@otago.ac.nz (P.S.); phil.bremer@otago.ac.nz (P.B.); 2 Riddet Institute, Private Bag 11 222, 4442 Palmerston North, New Zealand; 3 Department of Chemical and Materials Engineering, University of Auckland, Private Bag 92019, 1142 Auckland, New Zealand; m.farid@auckland.ac.nz

**Keywords:** frying, mathematical model, mass transfer, heat transfer, pulsed electric fields, solid plant foods

## Abstract

Deep-fried foods (e.g., French fries, potato/veggie crisps) are popular among consumers. Recently, there has been an increased interest in the application of Pulsed Electric Fields (PEF) technology as a pretreatment of plant-based foods prior to deep-frying to improve quality (e.g., lower browning tendency and oil uptake) and reduce production costs (e.g., better water and energy efficiencies). However, the influence of a PEF pretreatment on the frying process and related chemical reactions for food materials is still not fully understood. PEF treatment of plant tissue causes structural modifications, which are likely to influence heat, mass and momentum transfers, as well as altering the rate of chemical reactions, during the frying process. Detailed insights into the frying process in terms of heat, mass (water and oil) and momentum transfers are outlined, in conjunction with the development of Maillard reaction and starch gelatinisation during frying. These changes occur during frying and consequently will impact on oil uptake, moisture content, colour, texture and the amount of contaminants in the fried foods, as well as the fried oil, and hence, the effects of PEF pretreatment on these quality properties of a variety of fried plant-based foods are summarised. Different mathematical models to potentially describe the influence of PEF on the frying process of plant-based foods and to predict the quality parameters of fried foods produced from PEF-treated plant materials are addressed.

## 1. Introduction

Pulsed Electric Fields (PEF) technology applies short (μs or ms) and repetitive electric pulses of high voltage to food materials placed between two conducting electrodes, leading to electroporation of cells [1]. When plant tissues are exposed to PEF, the charging process increases the transmembrane potential leading to the breakdown of proteins and the lipid bilayer within the cell membrane. When the transmembrane potential exceeds the range that cells can withstand, the cell membrane is punctured followed by the formation of pores. Pores grow in both size and quantity depending on the intensity of PEF treatment. Critical parameters of PEF processing include the electric field strength (*E*), pulse frequency (*f*), pulse number (*N*), pulse shape and polarity, specific energy input (*W*), pulse width (*τ*) and duration (*t*) [2]. In recent years, PEF has been recognised as an effective technology to improve food quality and accelerate heat and mass transfers during food processing, while reducing energy consumption. PEF can be easily integrated into the food industry to assist existing unit operation, such as osmotic dehydration, freeze-drying, frying, freezing, thawing, extraction or clarification [3,4,5].

Frying (typically at 170 °C or above) is one of the oldest unit operations used by food processors; its goal is to produce final products with a crisp texture, an aromatic flavour and a golden-brown colour. There is a large variety of fried plant-based products available in the market, including fried vegetables such as potato, sweet potato, carrot, red beet, taro, celery bulb, squash, pumpkin, green bean and eggplant and fried fruits such as pineapple, apple, banana, peach, grape, guava, jamun and mango [6,7,8,9]. Among these, fried potato products (i.e., French fries and potato crisps) are the most widely consumed around the world [10]. Taking the production of French fries as an example, the process line consists of potato washing and sorting, skin peeling, preheating, cutting into fries, blanching, predrying, par frying and finally blast freezing. PEF is recommended to be applied as a pretreatment to the potatoes before the cutting step, potentially replacing the preheating step to reduce energy consumption while achieving equivalent process performance [11,12]. Under suitable operating parameters, PEF treatment will modify the structural and textural properties of potatoes, making them easier/more flexible to cut into fries, thereby reducing “feathering” and at the same time, extending the durability of the cutting blades [11,12]. With respect to the quality of the fried potato products, a lower browning tendency and a crispier texture compared to their non PEF-treated counterparts has been reported in the literature [11,12,13,14]. Such advantages offered by PEF in terms of process performance and product improvement make it appealing for the potato industry to adopt this technology.

Frying is a very complex process including simultaneous heat, mass and momentum transfers accompanied by a series of physical and chemical reactions [15,16]. During the frying process, heat is transferred from oil to fried foods leading to mass transfer (e.g., water evaporation and oil uptake). Apart from heat, mass and momentum transfers, chemical constituents (e.g., starch, reducing sugars, amino acids and water) within plant tissue react with each other during frying, and physical reactions (e.g., water evaporation and oil uptake) occur accompanied by structural changes [17]. Frying models, especially those built based on universal physical laws, may provide a better understanding of the frying process and its mechanism [15,18]. Additionally, the incorporation of observational data into kinetic frying models can give predictions of a specified quality of interest (e.g., colour, texture, oil uptake) for fried foods [19].

The purpose of this review is to provide an overview of the different mechanisms and reactions that occur during frying processes and explain how PEF pretreatment of plant materials can result in various quality improvements in the final products. The review will be concluded by discussing the potential use of frying models to explore the effect of PEF on frying to benefit further research and industry application.

## 2. Frying Process of Food Materials

**Figure 1:**
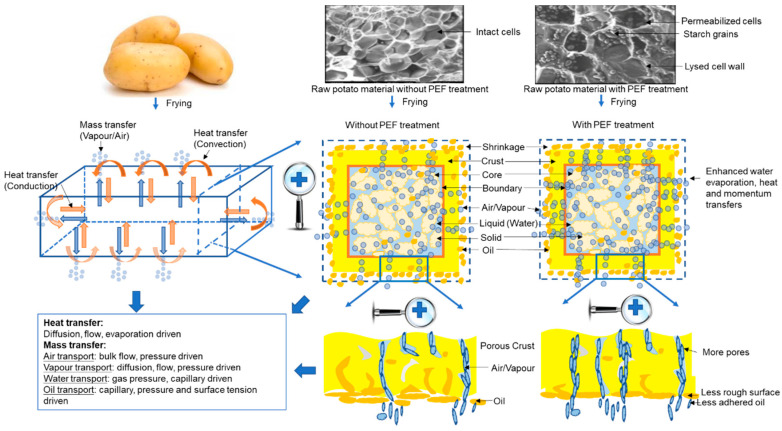
Potential influence of the Pulsed Electric Fields (PEF) pretreatment of potatoes on heat and mass transfer processes during frying. Images of untreated and PEF-treated raw potato tissue from scanning electron cryomicroscopy (cryo-SEM) were obtained from [28].

Frying is a complex process which involves simultaneous heat, mass and momentum transfers (Figure 1), resulting in the flow of oil and water, phase changes and physicochemical reactions within the raw materials [17]. These reactions account for both the beneficial and the deleterious effects associated with fried foods.

### 2.1. Heat Transfer

Frying is an efficient and intensive heat transfer process owing to the high heat transfer coefficients and dynamic conditions within the frying system [20]. During frying, heat transferred from the hot oil to the surface of the cold food materials is driven by *convection* and from the surface to the inner side by *conduction* [18]. Then, water begins to evaporate from the food and creates vapour turbulence because of its rapid evaporation. Heat is transferred by forced convection contributing to the turbulence of the oil around the food [21]. Vapour turbulence enhances the heat transfer rate to its maximum level [22]. When large numbers of vapour bubbles cannot escape from the food material, they form an insulating layer on the surface limiting heat transfer [23]. During frying, water evaporation dissipates some energy inside the food material, thereby decreasing the available energy for temperature increase. Normally, the change of energy for heat transfer within a nonhomogenous food material can be calculated using the following Equation (1) in Cartesian coordinates [24]:

(1) $${\;\rho C}_{p}\left(\frac{\partial T}{\partial t}{+v}_{x}\frac{\partial T}{\partial x}{+v}_{y}\frac{\partial T}{\partial y}{+v}_{z}\frac{\partial T}{\partial z}\right){=k}_{h}\left(\frac{\partial^{2}T}{\partial^{2}x}+\frac{\partial^{2}T}{\partial^{2}y}+\frac{\partial^{2}T}{\partial^{2}z}\right)$$

*T*: temperature, *C_p_*: specific heat capacity of the material, *k_h_*: thermal conductivity of the material, *ρ*: density, *v_i_*: fluid velocity in *i*-direction. The right side of Equation (1) represents heat transfer due to conduction (dominates in case of solids) while the left side of the equation represents the unsteady term and convective heat-transfer terms.

In terms of frying, the energy balance equation is as follows [24]:

(2) $${\rho C}_{p}\frac{\partial T}{\partial t}=\frac{\partial }{\partial x}\;k_{eff}\;\frac{\partial T}{\partial x}+\frac{\partial }{\partial y}\;k_{eff}\;\frac{\partial T}{\partial y}+\frac{\partial }{\partial z}\;k_{eff}\;\frac{\partial T}{\partial z}+\Delta H^{sbl}\;w$$

*k_eff_*: effective thermal conductivity, Δ*H^sbl^*: latent heat of sublimation, *w*: water content.

There is a lack of information on the influence of PEF on heat transfer of food materials during frying, but current literature has demonstrated that PEF technology can accelerate the heat transfer during drying and osmotic dehydration processes [5]. Since the heat transfer coefficient increases with the rate of moisture transfer [25,26], an increase in tissue porosity and cell electroporation by PEF may increase the hydrodynamic permeability and the moisture transfer rate [27]. Therefore, the heat transfer and efficiency of drying or frying of PEF-treated plant materials is expected to be enhanced.

### 2.2. Mass Transfer

During the frying process, mass transfer mainly refers to the evaporation of water from the food material to the oil as well as the uptake of oil by food material [29]. Previous studies have demonstrated that PEF treatment enhances mass transfer in plant-based foods compared to non PEF-treated materials due to its electroporation effect on cell membranes, and subsequently this improves the diffusion of intracellular liquid/cell contents to the outside of the cells. [3]. Ignat et al. [13] have reported that PEF-treated potato cubes had a significantly higher drip loss (10.4%) compared to their non PEF-treated counterpart (2.3%) before frying. This phenomenon occurs due to PEF causing structural changes in the potato tissues which increase drip loss and facilitate the release of sugars which lowers the tendency for the tissues to brown during frying [13]. Moreover, PEF-treated potatoes exhibited a faster rate of water loss compared to non PEF-treated samples after 10 min baking at 100 °C [30]. Therefore, PEF pretreatment has the ability to enhance water diffusion from plant materials, which could promote a faster frying efficiency.

#### 2.2.1. Water Transfer

There are four main stages in the frying process which involve heat and mass transfers, namely the initial heating, surface boiling, falling rate and bubble endpoint periods [21,31]. In the *initial heating* stage, raw materials (at cold or ambient temperature) are dropped into the hot oil and are heated up gradually to the boiling point of water. During the *surface boiling* period, water begins to evaporate from the surface of materials along with the formation and release of bubbles leading to a rapid loss of water and the formation of pores at the surface becomes inevitable. A crust also begins to form at the outer surface. In the *falling rate* stage, the humid core region of the food material is heated slowly to the boiling point of water. Meanwhile, crust thickness increases and steam transfer speed decreases during this stage. In the *bubble endpoint* stage, water evaporation slows until bubbles are no longer being released on the surface.

Water transfer in the form of liquid (*C_l_*) and vapour (*C_v_*) during the unsteady diffusion process at the initial heating stage can be represented by the following equations in Cartesian coordinates [24]:

(3) $$\frac{\partial C_{l}}{\partial t}=\frac{\partial }{\partial x}\;D_{i\;}\frac{\partial C_{l}}{\partial x}+\frac{\partial }{\partial y}\;D_{i\;}\frac{\partial C_{l}}{\partial y}+\frac{\partial }{\partial z}\;D_{i\;}\frac{\partial C_{l}}{\partial z}\;-\;w$$

(4) $$\frac{\partial C_{v}}{\partial t}=\frac{\partial }{\partial x}\;D_{i}\;\frac{\partial C_{v}}{\partial x}+\frac{\partial }{\partial y}\;D_{i}\;\frac{\partial C_{v}}{\partial y}+\frac{\partial }{\partial z}\;D_{i\;}\frac{\partial C_{v}}{\partial z}+w$$

*C_i_*: concentration of liquid (*i* = *l*) or vapour (*i* = *v*), *D_i_*: diffusivity of *i-*th species in the medium, *w*: water content.

During frying, the food material is transformed into a porous medium consisting of tiny void spaces (or small pores) that are interconnected and filled with fluid (liquid or vapour) [32]. Therefore, the movement of water vapour can no longer be described as diffusion. Darcy’s law is considered to be more appropriate in describing the flow of water vapour inside the solid through the porous structure of fried foods [24]. Since plant materials typically contain a high water content (80–95%), Darcy’s equation takes into account the significant pressure build up inside the porous food caused by the evaporation of internal water during frying [32]. Moreover, the resistance of the porous structure, which is proportional to the thickness of fried foods, can be integrated into Darcy’s law formulating Equation (5) to better describe water loss due to vapour flow through the crust [24].

(5) $$Q_{w}=\frac{A\;k\;\Delta P}{\mu \;L}$$

*Q_w_*: flow rate of water vapour, *A*: cross-sectional area of the fried food, *k*: permeability of the crust layer (related to porosity), Δ*P*: pressure difference/drop over a given distance, *μ*: viscosity of the water vapour, *L*: thickness of fried foods.

#### 2.2.2. Oil Transfer

There are several mechanisms to explain oil uptake during frying including w*ater replacement*, *capillarity penetration*, the *cooling-phase effect* and the *surface-active agent theory*, all of which are associated with water transfer and/or crust formation.

*Water replacement mechanism* explains that oil enters the plant tissues through the voids created by water evaporation, so water loss is considered to be the basis of oil uptake [33]. Gamble et al. [34] found that oil uptake was closely related to the water loss (R^2^ = 0.989) in potato slices in which the oil absorbed by potato slices was found accumulated in the voids left by the water evaporation. When the water bubbles escaped from food materials, they formed capillary pathways and increased the surface porosity [35].

*Capillarity penetration* describes how the oil moves upwards through narrow pores in fried materials when the adhesive intermolecular forces between the oil and food materials are stronger than the cohesive intermolecular forces in the oil [36]. The pressure difference (ΔP^*^) between both ends of the capillary pathways mainly drives the capillarity penetration phenomena [37]:

(6) $$\Delta P^{*}{=P}_{2\;}{-\;P}_{1\;}{=P}_{atm}\;{-\;(P}_{v}\;-\;\frac{2\sigma \;cos\theta }{r}\;\pm \;\rho gh\;cos\alpha )$$

*P_i_*: pressure at the point *i* (*P_2_:* pressure at the pore surface, *P_1_:* pressure at the deepest pore point inside the food material, *P_atm_:* atmospheric pressure, *P_v_:* water vapour pressure), *θ*: contact angle between the oil and the food material, *r*: pore radius, *σ*: surface tension of the oil, *ρ*: oil density, *g*: acceleration gravity, *h*: height of the capillary motion, *α*: angle between the capillary pathway and vertical direction.

Capillary penetration can also be described by the Washburn equation:

(7) $$Q_{oil}=\frac{{\;\pi r\;}^{4\;}\Delta P^{*}}{8\;\mu \;h}$$

*Q_oil_*: volumetric flow of laminar oil, π: ratio of an oil circumference to its diameter (3.142), *r*: pore radius, *μ*: oil viscosity.

The penetration of oil over time can be calculated based on the modification of the above two Equations (6) and (7) to yield Equation (8) [37]:

(8) $$\frac{dh}{dt}=\frac{r^{2}}{8\mu \;h}{\;(P}_{atm\;}{-\;P}_{v\;}+\frac{\;2\;\sigma \;cos\theta }{r}\;\pm \;\rho \;g\;h\;cos\alpha )$$

However, the voids or capillary pathways are always filled with water during frying and the inner steam pressure may resist oil penetration. Sometimes, the oil is absorbed after the capillary (food material) is removed from the oil [35,37].

The *mechanism of cooling-phase effect* explains oil uptake after the food material has been removed from the oil and this is caused by water vapour condensation and internal pressure reduction during the cooling period. A study by Ufheil and Escher [38] reported that most of the oil in fried potatoes is absorbed into the porous crust after their removal from the oil, implying that oil absorption and water loss are not synchronous and that the cooling-phase effect plays a key role in oil uptake. Oil uptake after removal of the plant tissue from the oil is a balance between the oil drainage and oil adhesion [17]. Adhered oil on the surface of fried material is absorbed due to the “vacuum effect” caused by the condensation of steam during cooling period. When the fried food is removed from the oil, an oil film is formed on the surface and its thickness (*H*) can been calculated by the Landau–Levich–Derjaguin equation [39]:

(9) $$H=0.944\;\frac{{\left(\mu U\right)}^{\frac{2}{3}}}{{\gamma \;}^{\frac{1}{6}}{\left(\rho \;g\right)}^{\frac{1}{2}}}$$

*μ*: oil viscosity, *γ*: surface tension, *U*: speed of oil removal after frying; *ρ*: oil density, *g*: acceleration gravity.

Finally, the *surface-active agent theory* has been proposed in addition to the other oil uptake mechanisms. In this theory surface-active agents (e.g., monoglycerides and diglycerides) produced by oil degradation and hydrolytic reactions during frying enhance the interactions between oil and fried food leading to increased oil absorption [35]. Such surface-active agents can increase the foaming tendency of oil and reduce the interfacial tension leading to the increase of surface hydrophobicity [40].

### 2.3. Momentum Transfer

Momentum transfer is a physical phenomenon that involves convection mechanism between molecules or groups of molecules within the food material [41]. It depends upon the interrelation of the fundamental variables of mass, velocity and time and of changes in the velocity per unit mass [41]. During frying, momentum is transferred by *convection* (vapour leaving the fried materials) or by *molecular forces* (viscous stress or pressure) [42]. Momentum transfer equations are based on the principle that the momentum is conserved in a phase. The momentum balance equation, which contains three velocity components and the *x*-component equation in Cartesian coordinates [15,43] is as follows:

(10) $$\;\rho \;\left(\frac{\partial v_{x}}{\partial t}{+v}_{x\;}\frac{\partial v_{x}}{\partial x}{+v}_{y\;}\frac{\partial v_{x}}{\partial y}{+v}_{z\;}\frac{\partial v_{x}}{\partial z}\right)=\left(\frac{\partial }{\partial x}\mu \;\frac{\partial v_{x}}{\partial x}+\frac{\partial }{\partial y}\mu \;\frac{\partial v_{x}}{\partial y}+\frac{\partial }{\partial z}\mu \;\frac{\partial v_{x}}{\partial z}\right){+\rho \;g}_{x}{\;\beta \;(T\;-\;T}_{\infty })$$

*v_i_*: fluid velocity of *i* component, *ρ*: fluid density, *g_x_*: acceleration gravity of *x*-component, *β*: thermal expansion coefficient.

Despite there being no studies directly reporting the influence of PEF pretreatment on momentum transfer in plant materials during frying, Dellarosa et al. [44] have shown that PEF could help to gain momentum to increase mass transfer in plant-based foods during processing, which suggests that the momentum transfer in PEF-treated plants would increase during frying.

## 3. Changes in the Chemical Constituents of Plant Food Material during Frying

Plant foods are a good source of carbohydrates (e.g., simple sugars, starch) and proteins. Frying of plant foods at high temperatures (>170 °C) initiates a series of complex chemical reactions in these constituents when reacting with oil, and thus generating compounds that can affect the quality of final products, by influencing their flavour, colour, shelf life and nutrient composition [45]. The two chemical changes that this review will focus upon are Maillard reaction and starch gelatinisation.

### 3.1. Maillard Reaction

The Maillard reaction is one of the most important chemical reactions that occur during frying because it modifies many quality parameters in the final fried product such as colour, flavour, taste, nutritional value and the level of toxic compounds (e.g., acrylamide) [46]. The Maillard reaction refers to the reaction between an amino group (e.g., amino acids) and a carbonyl group (e.g., reducing sugars). Firstly, a Schiff base is formed and rearranged to Amadori or Heyns products, which then undergo enolisation and are subsequently modified to form reactive α-dicarbonyl compounds, the source of brown pigments production [46,47]. These compounds can react with additional nucleophiles (i.e., guanidines, amines and thiols) and undergo Strecker degradation producing Strecker aldehydes. Furthermore, advanced glycation end products are produced in a series of downstream reactions, and further chemical reactions form a large number of polymerised products, named melanoidins, which result in colour darkening [46]. Temperature, time, reactant type (amino and carbonyl groups) and concentration, water activity and pH are the main factors that influence the Maillard reaction [48]. Most reactants, primarily reducing sugars and amino acids involved in the Maillard reaction are accumulated in vacuoles in the plant’s tissues. Since PEF treatment induces a cell electroporation effect on plant tissues which results in leakage, it is not surprising that a considerable amount of the reactants might be released/leached out from the cells, as demonstrated in the potato studies by Janositz et al. [30] and Genovese et al. [49]. An increase in the release of reactant materials from PEF treated potatoes is generally beneficial for high sugar containing potatoes (due to genotype, maturity, and poor storage management), which can exhibit excessive browning when fried. This is because the overall pool size of the reactants in these potato tissues, after a PEF-pretreatment, that is available to participate in the Maillard reaction and caramelisation during frying can be reduced.

### 3.2. Starch Swelling and Gelatinisation

Potato tubers are high in starch, which is a semicrystalline biopolymer. When potatoes are cooked, starch starts to swell and its gelatinisation forms dense and starch-rich areas within the cells, which helps to reduces oil absorption upon frying and resist dehydration and shrinkage, while improving the texture of the cooked products [35]. Starch gelatinisation refers to the collapse of molecular organisation in the starch granule, leading to irreversible changes to its molecular properties resulting in water uptake, swelling, loss of crystallinity, loss of birefringence, unwinding of double helices, starch solubilisation and an increase in viscosity [50]. During frying, starch swelling begins at 60–70 °C and the process is completed when the swollen starch granules completely occupy the interior of the cells, which enable them to resist oil penetration [51,52]. Moreover, the extent of starch gelatinisation in the crust and core regions of potato crisps can be very different owing to different rates of heat transfer and water availability [53].

Numerous studies investigating the effect of PEF on starch isolated from different plant sources, have consistently demonstrated that the application of a high intensity PEF treatment (30–50 kV/cm) can alter starch structure and its inherent properties [54,55,56]. Under the influence of PEF, the crystalline region and the side chains of the amylopectin in the starch can be modified leading to a decrease in the double helix binding force and disruptions to the intragranular molecular arrangement of starch granules. Such changes in the starch promote reactions between water molecules and starch chains, reducing the energy required for starch gelatinisation. Starch gelatinisation temperature and enthalpy of gelatinisation were found to decrease with an increase in PEF intensity [54], suggesting that the starch may become more susceptible to gelatinisation after PEF treatment.

## 4. An Overview of the Quality Parameters of Fried PEF-Treated Plant Food Materials

While it is clear that PEF pretreatment of plant materials can influence a range of reactions that occur during frying, including altering the heat and mass transfers, Maillard reaction and starch gelatinisation, all of these processes are expected to influence the quality of the final products. Table 1 summarises how PEF treatment can affect the quality parameters (e.g., colour, moisture content, oil uptake, texture and toxic compound) of plant-based foods, specifically on potato, during frying.

### 4.1. Colour

Colour is considered the most important parameter contributing to the visual perception of the quality of foods, and it influences the acceptance and choice of consumers. Apart from Maillard reactions, oil degradation may also affect the colour of fried foods. For example, a high correlation (R^2^ > 0.9) has been reported between the dark colour of fried tortilla chips and oil degradation time [57]. The polymerisation of triglycerides and the products of triglyceride hydrolysis, such as free fatty acids, monoglycerides and diglycerides, during oil degradation may result in changes in colour [57]. Process variables including raw material properties (i.e., reducing sugar content, amino acids content, protein content and dimensions), frying temperature, time and oil type can also affect the colour of fried food [58].

The effect of PEF treatment on the colour changes of different types of fried plant-based foods has been reported. Specifically considering potatoes, studies by Ignat et al. [13] and Genovese et al. [49] have both reported that the final products produced from PEF-treated potatoes are less brown with a uniform and bright colour after frying. A similar finding was also observed in fried chips produced from PEF-treated sweet potato [19].

### 4.2. Moisture Content

When a plant material is placed into hot oil, its surface temperature increases rapidly and surface water rapidly evaporates in the form of water bubbles when its temperature rises to 100 °C [21]. As a result, the surface dries quickly and forms a crust. This crust acts as an additional barrier to the escape of water from the inner regions; therefore, the inner region is always moist compared to the outer region [60]. A number of other factors can also affect the moisture content of fried foods such as potato crisps, including frying temperature and time, the size and shape of the product and prefrying procedures such as drying [61].

PEF treatment prior to frying can help to accelerate moisture loss from plant materials during frying based on the results gathered from previous studies [14,30,61]. The effect of fast moisture loss from PEF-treated food material on the hydrolysis of oil can lead to deterioration to frying life of the oil, and hence further research in this aspect is recommended. However, moisture evaporation from fried food can also form a “steam blanket” (or physical barrier) on the surface of the oil and thus prevent the contact between atmospheric oxygen and oil, limiting the oxidation of frying oil [62].

Moreover, a faster reduction in water content has been observed in PEF-treated potato discs/slices during air-drying process followed by frying compared to untreated potato samples [14,59]. Such findings indicate that PEF could be used to enhance/facilitate the overall drying and frying efficiency through accelerating the moisture diffusivity and water loss owing to the cell electroporation effect on plant cell membrane, which results in a greater diffusion of intracellular water out of the cells. However, when most of the surface water within a potato tissue has been evaporated during frying, the influence of PEF on water loss may become insignificant [59].

### 4.3. Oil Uptake

Oil is the most important ingredient for frying since it drives heat and mass transfer during frying. As discussed in Section 2.2.2, most of the oil is absorbed by entering pores through which moisture escapes during frying. Factors, such as frying conditions (time, temperature, food-to-oil ratio, repeated frying), oil characteristics (quality and type) and food characteristics (size, shape, surface roughness and porosity) and pretreatments (blanching, edible coating, vacuum drying, PEF, etc.) may influence the amount of oil absorbed [63].

Both Liu et al. [14] and Janositz et al. [30] have reported that the oil uptake for PEF-treated potato slices (1.5–2.5 mm thickness), after frying, was between 34 and 39% lower compared to untreated samples. Therefore, PEF is an effective pretreatment option for solid plant materials in order to reduce oil uptake during frying and may have implications on the management of frying oil and the quality of fried foods. There are several possible reasons to explain this phenomenon. Firstly, PEF treatment appears to increase the porosity of plant tissue leading to a higher vapour pressure and faster vapour movement to the surface, thus limiting the oil absorption [30]. Secondly, a smoother cut surface (less “feathering”) of PEF-treated plant material results in a significant reduction in the amount of adhered oil after frying [19]. Thirdly, since PEF pretreatment can hasten starch gelatinisation during frying, this promotes the formation of an impermeable surface layer, which consequently acts to inhibit oil absorption [14]. Another likely phenomenon could be the cell electroporation effect of PEF in conjunction with an increase in the formation of capillaries resulting in an increased tendency for the oil to leach out from the pores/capillaries and thus leave the surface of the fried food.

### 4.4. Texture

Fried foods are expected to have a crispy crust and a moist and soft interior [17]. Crispness, porosity and shrinkage are regarded as the main textural indicators of fried foods.

#### 4.4.1. Crispness

Crispness describes a quick fracture under small strain stresses owing to a low water content in the surface layer of a thick fried piece or through a thin slice [17]. It is important to note that the effect of PEF treatment on the crispness of fried foods cannot be assumed since this textural parameter is highly dependent on the par-frying time and temperature, oil content, moisture content, starch content, porosity and roughness of surface [11,61]. Cahayadi et al. [64] reported that fried crisps produced from PEF-treated potatoes were perceived to be crunchier compared to potato crisps from untreated potatoes, and this texture modification of the potato crisps has shown to increase the perceived satiation of an individual and thereby reduce energy intake from snack consumption.

An important factor contributing towards the texture of French fries is their starch content and therefore excessive starch loss during the production of French fries should be avoided [11]. While it has been recognised that PEF treatment can induce the formation of irreversible pores in cell membranes causing leakage of cell contents (e.g., free sugars and amino acids), this electroporation effect on plant tissue may not necessary impact severely on the leakage of polymers such as starch. The retention of starch has been observed in PEF-treated potatoes subjected to industrial scale French fries production [12]. In fact, potato starch granules with an average diameter of 40 μm, which possibly became larger after PEF treatment [55], were not able to pass through the PEF-induced membrane pores that had a maximum size of around 5 μm [65].

#### 4.4.2. Porosity

During frying, some water vapour is unable to move through the food material because of restrictive intercellular diffusion. As a result, superheated vapour distorts the pores and leads to the formation of a porous structure [66]. Normally, intense water evaporation results in the formation of more pores that are larger [66]. There are many factors influencing the porosity of fried foods, including frying conditions (time, temperature, and pretreatments), plant material characteristics (size, shape, component, density) and oil types. For example, it has been reported that the porosity of fried foods increased with increasing oil temperature along with the use of hydrogenated oil [66].

Though the effect of PEF on the porosity of fried foods is yet to be reported in the literature, it is reasonable to expect that PEF treatment may increase the porosity of fried foods according to the observations from PEF-assisted drying process. An increase in surface porosity was found in air-dried products (e.g., apple cubic slabs, apple slices and potato slices) due to a PEF pretreatment [16]. This is likely to occur owing to enhanced heat and mass transfer rates caused by PEF, resulting in an intense water evaporation and thus, an increase in the size and number of pores.

#### 4.4.3. Shrinkage

Shrinkage is caused by water loss, resulting in the reduction of open pores and an increase in the density of fried products [61]. During frying, shrinkage initially occurs at the surface accompanied by the formation of a rigid outer layer. Shrinkage then moves inwards until the final volume is fixed. Shrinkage phenomena are related to the frying time and temperature, shape, size and density of the food materials [61]. For example, it has been reported in fried sweet potatoes that shrinkage is more pronounced with increased frying time and higher frying temperatures [67].

No research has been published studying the influence of PEF on the shrinkage of fried foods, but previous research on PEF-assisted drying process has suggested that PEF pretreatment could lead to product shrinkage. Air-dried products from PEF-treated carrots and red beetroots shrink more compared to non-PEF treated samples owing to tissue damage caused by the cell electroporation effect [27,68].

### 4.5. Toxic Compounds

Toxic compounds that may be produced during frying include acrylamide, hydroxymethylfurfural, furan, ethylcarbamate, heterocyclic amines, polycyclic aromatic hydrocarbons and nitrosamines, all of which are potential health risks to consumers [69]. The presence of acrylamide in fried potato products (typically an average of 400 ng/g, where the upper recommended limit by the European Commission is 1000 ng/g) is unavoidable [70] due to the high concentration of acrylamide precursors, reducing sugars and asparagine in potato tubers [71]. Acrylamide has genotoxic, neurotoxic and carcinogenic risks in animal [72]. Most acrylamide in fried foods are formed by the Maillard reaction between reactive carbonyls and asparagine at a temperature of over 120 °C through a series of intermediates [20]. Factors such as frying conditions (temperature, time, pH and pretreatment) and material characteristics (cultivar, chemical constituents, water activity) may influence the acrylamide content in fried plant-based foods [20].

A recent finding by Genovese et al. [49] was that the acrylamide content of potato crisps produced from PEF-treated potatoes was about 30% lower than those for untreated crisps. PEF may considerably reduce, through diffusion, the reactant content available to participate in the Maillard reaction and hence the formation of toxic compounds during frying is inhibited.

## 5. The Use of Frying Models to Describe the Frying Process of PEF-Treated Plant Materials

**Figure 2:**
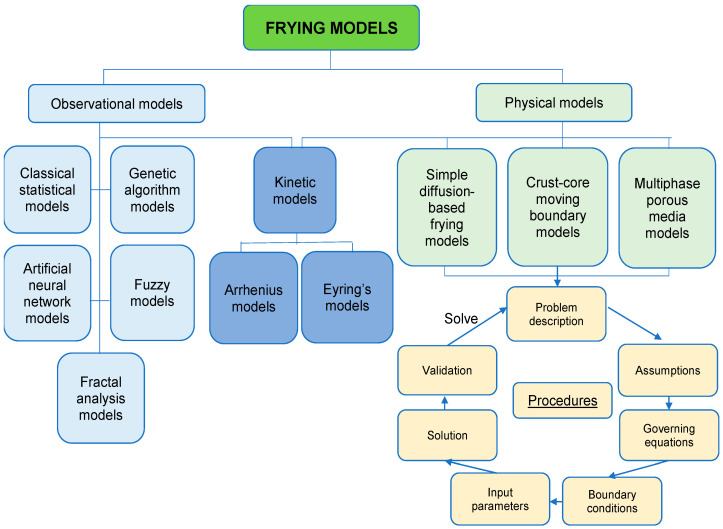
Summary of mathematical models suitable to describe frying process and the model building process.

Different frying models have been proposed in the literature (Figure 2). *Physical models* are built based on universal physical laws and the underlying mechanisms behind frying process, so their predictions are usually more precise and based on fundamental physical phenomena. *Observational models* are built based on the fitting of experimental data, so they are also known as data-driven models. *Kinetic models* are built to describe the rates of chemical reactions relevant to some universal physical laws by fitting experimental data into the model. Therefore, kinetic models are classified under both physical and observational models [73]. In this review, selected frying models will be briefly discussed and then evaluated regarding their suitability to be used in describing the influence of PEF treatment on the frying process of plant-based foods.

### 5.1. Physical Models to Describe Heat, Mass and Momentum Transfers

Physical frying models require equations that describe changes in mass, heat and momentum transfers, and simultaneously considering the phase changes and physicochemical changes of plant-based foods during frying. They can provide an in-depth understanding of the physical process of frying, and they are usually more precise because the models are built based on the universal laws. Most physical models for frying are macroscopic continuum models of heat, mass and momentum transfers [73]. However, the coordinate systems, suitable equations and boundary conditions may vary between different frying conditions. Because of the complexity of frying process, different types of physical models have been built (Table 2). These can be divided into *simple diffusion-based*, *crust-core moving boundary* and *multiphase porous media* models.

The procedures of building physical models for the frying process are straightforward (Figure 2). The first step is to define the purpose and describe the questions to be solved with this model. Then, some assumptions (about shapes, geometrical dimensions, mass or heat transfer coefficients, material properties and volume changes) are usually made to simplify the complex real-life situation [74]. The governing equations for heat, mass and momentum transfers are the core of theoretical models and the typical equations vary between different types of models (Table 3). *Heat transfer* is usually modelled by conservation of heat equation and Fourier’s equation, *mass transfer* is generally modelled by conservation of mass equation and Fick’s law of diffusion and *momentum* transfer is always described by conservation of momentum equation and Navier–Stokes equation [74]. Each governing equation has its boundary conditions, which reflect the interaction between the material being fried and surroundings, in order to describe the frying process accurately. Knowledge of boundary conditions occurring during the frying process are required to solve these equations numerically. The commonly used methods to obtain solutions for the boundary conditions include the finite difference, finite element, finite volume, boundary elements, lattice gas cellular automata and lattice Boltzman methods, and many of them rely on the commercial computational software, such as the computational fluid dynamics and computer aided food process engineering. Then input parameters such as density, specific heat capacity, thermal conductivity and permeability are introduced to the model. After building the theoretical model, experimental data from frying process is used to verify the model.

The *simple diffusion-based frying model* is the simplest physical model to describe frying. It is built based on the simple heat conduction and moisture diffusion processes while ignoring the oil absorption and water evaporation altogether in the fried material. Rice and Gamble [75] attempted to build a one-dimensional water diffusion model combining both Fick’s first law and Arrhenius relationship to predict the moisture loss during the frying process of potato slices and successfully proved that the model made valid predictions regarding the early stage of frying (within the first 180 s). Likewise, Pedreschi et al. [76] applied Fick’s law of diffusion with constant and variable effective diffusion coefficients in order to model the water loss during frying. They found that a simple diffusion-based frying model that considered the change of diffusivity coefficient value with frying time was relatively more precise (better fit to experimental data) in predicting the moisture content of potato slices compared to a classic model with a constant effective moisture diffusion coefficient. However, it is important to note that simple diffusion-based frying models are only able to provide a limited understanding of the frying process because the complex pressure driven flow is simplified to effective diffusion and empirical parameters, which influences their accuracy and restricts the application of these models for different plant materials and frying conditions [77].

The *crust-core moving boundary model* is built based on the core and the crust regions formed in the food material during the frying process, taking into account the moving boundary, where the interface between the core and crust regions moves [31]. This model is expected to be more precise than the simple diffusion-based frying model because it considers the diffusional and pressure driven transports as well as the distributed evaporation. For example, a good agreement was found between the experimental data (water content, centre temperature, surface temperature and crust thickness) and predicted values using a one-dimensional moving boundary model that included pressure-driven flow, albeit ignoring diffusion flow in the crust region [78]. Other types of crust-core moving boundary models have been built dealing with different conditions. Acknowledging the temperature difference at different positions of a fried food, Southern et al. [79] developed a moving boundary model using the Fourier’s law and energy balance equation to describe heat transfer in both the core and crust regions and significantly improved the theoretical prediction of the experimental temperature-time values in different locations of potato crisps during frying. Moreover, van Koerten et al. [80] built a crust-core moving boundary model based on a Nusselt correlation connecting heat transfer coefficient and water evaporation rate, which was demonstrated to be a simple but effective model for predicting water evaporation and temperature profile in potato cylinders of different diameters (8.5, 10.5 and 14 mm). To allow the models to be applied more widely for different conditions, Farid and Kizilel [42] developed a unified moving boundary model, by defining a parameter which could reflect the extent of mass diffusion relative to thermal diffusion, to predict the temperature and moisture distribution in a food material, which can be applied in any drying and frying processes. The unified model successfully described the temperature and moisture distributions during the frying and air-drying process for thick (25.4 mm) and thin (2–3 mm) potato slices. Thus, crust-core moving boundary model can be a suitable model to describe the frying process regardless of the dimensions and positions of the fried materials. However, the rate of heat transfer during frying process is highly dependent on the food properties, such as the thermal conductivity and water diffusivity and hence, they should be considered in the mathematical model [81]. In addition, analytical solutions for complicated equations and boundary conditions are sometimes unavailable. Application of PEF to plant materials is expected to alter the microstructure and physicochemical properties (e.g., thermal conductivity and water diffusivity), hence influencing the moving boundary of crust-core regions. Therefore, crust-core moving boundary model could be considered in future research to describe the differences of frying process between untreated and PEF-treated plant-based foods in a simple yet precise manner.

The *multiphase porous media model* is built based on the simultaneous heat, mass and momentum transfers. Multiphase transport in a porous media can be due to three underlying mechanisms including the molecular diffusion for gases, capillary diffusion for liquids and convection (pressure driven or Darcy flow for liquids or gases) [32]. The multiphase porous media model is considered more realistic, comprehensive and can provide better insight into frying process because it includes the temporal and spatial profiles of temperature and the transport mechanisms of water (liquid and vapour) and air inside the fried materials [82,83]. Researchers have applied the multiphase porous media model for different frying conditions, and hence, are able to explain the frying process from multiple perspectives. For example, Ni and Datta [84] developed a multiphase porous media model to predict the temperature, moisture, oil uptake and crust thickness of potato slices that took into consideration the pressure driven flow for the oil, vapour and air phase in the porous medium. This model shows that there is a pseudo-steady state region in the dry crust and a transient diffusion-like profile in the wet core but it becomes spatially uniform with frying time. Similarly, Halder et al. [77] have also developed a multiphase porous media model for potato frying and postfrying cooling process based on the nonequilibrium equation for evaporation. The estimated heat and mass transfer coefficients accurately reflect the process of different phases including the nonboiling phase and surface boiling and falling rate stages in the boiling phase. As a result, there is a reasonably good agreement between the experimental data and predicted values of quality parameters, such as the oil content, crust thickness and acrylamide content and this model can be applied to describe baking, meat cooking and drying processes with minor modifications. Multiphase porous media model can be applied to vacuum frying, where Warning et al. [85] have developed a model of potato chips by modifying the Darcy’s law to account for the Klinkenberg effect. It works well to predict the moisture, temperature, pressure, oil content and acrylamide content during vacuum frying and it implies that the core pressure is approximately 40 kPa higher than the surface pressure of the potato chips. While it is clear that multiphase porous media models are capable of describing the multifaceted physics behind frying process, some of them are difficult to implement in real-life scenario due to the complexity of calculating the evaporation rate. Application of PEF to plant materials is expected to influence the porosity of raw materials. Therefore, multiphase porous media model could be suitable to describe the frying process of PEF-treated plant materials since the model can describe the heat and mass transfers inside a porous material in conjunction with the air flow outside the porous material, and phase changes such as evaporation and condensation [86].

### 5.2. Observational Models for Quality Prediction of PEF-Treated Fried Foods

Observational models are useful in building a relationship between the input and output parameters, especially when the practical situations are too complex to understand and building a physical model becomes unrealistic. When building observational models, assumptions and physical interpretations are not necessary since only experimental data are needed to fit a mathematical equation. Commonly used observational models includes *classical statistical*, *artificial neural network*, *genetic algorithm*, *fuzzy* and *fractal analysis* models [73]. Observational models are particularly useful to help optimise the frying process in order to obtain high-quality fried products. For example, statistical and two-stage fuzzy models have been built to optimize the blanching and frying parameters (e.g., oil temperature, thermal power) resulting in quality improvements of fried foods and efficiency of frying process [92,93]. Artificial neural network models have become popular in recent years and are considered to provide an accurate quality prediction. These models are built by selecting proper network structures and tuning model parameters such as weight, connections and threshold values until the fit to the experimental data is maximised. Mohebbi et al. [91] built a genetic algorithm-artificial neural network model which could predict the moisture and oil content of fried mushroom accurately. However, the predictive results from observational models can be significantly affected when the physical properties or environmental conditions are altered [77]. To study the influence of PEF on some reactions (e.g., Maillard reaction, starch gelatinisation, etc.) and quality parameters (colour, acrylamide content, etc.) of fried foods during frying process, observational models would be suitable because they can predict the results directly without understanding the complex reaction process.

### 5.3. Kinetic Models for Predicting the Rate of Frying Reactions after PEF Treatment

The quality of fried foods changes with frying time owing to chemical and physical reactions during the frying process. The changes of food composition and quality parameters are always described by the kinetic rate of frying reactions, which may also influence the heat and mass transfers [81]. The chemical, physical and biochemical changes such as degradation and formation of substances (amino acid, sugar, acrylamide, etc.), texture degradation and starch gelatinisation can be evaluated by kinetic models [94,95]. A clearer understanding of molecular level changes during the frying process can be obtained from kinetic models because they contain characteristic kinetic parameters such as rate constants and activation energies [96]. The frying reactions for PEF-treated plant materials can be influenced by the treatment intensity, and accordingly, alter the kinetic parameters of the chemical, physical and biochemical reactions occurring during frying.

The basis of a kinetic model for predicting quality changes is as follows:

(11) $$\frac{dP}{dt}{=\pm \;k\;P}^{n}$$

*P*: quality parameters, *k*: rate constant, *t*: time, *n*: reaction order.

Normally, reaction orders can be determined from the characteristics of a chemical reaction. The reaction orders may vary under different circumstances due to the complicated nature of food systems. As reported in the literature, the time dependency of chemical reactions and quality changes of texture, colour and nutrient content usually follows a zero or first order of reaction [97,98].

The temperature dependency of the rate constant (*k*) can be modelled using Arrhenius equation and Eyring’s absolute reaction rate theory model. The Arrhenius model is very common in the food industry for quality prediction, which can be written as:

(12) $$k=A{\;\exp}^{\left(\frac{-Ea}{RT}\right)}$$

*A*: pre-exponential factor, *E_a_*: activation energy, *R*: ideal gas constant (8.3136 J/molK), *T*: absolute temperature (K).

As for the Eyring’s absolute reaction rate theory, it is formed based on the transition state theory:

(13) $$A+B\;\overset{k_{1}}{↔}{\;AC}^{+}\;\overset{k_{2}}{\to }\;C$$

*A* and *B*: molecules, *AC+*: activated complex or transition state, *C*: product.

The Eyring equation is as follows:

(14) $$k=\frac{k_{b\;}T}{h}\;\exp{\;}^{\left(\frac{\Delta S^{+}}{R}-\frac{\Delta H^{+}}{RT}\right)}$$

Δ*S^+^*: activation entropy, Δ*H^+^*: activation enthalpy *k_b_*: Boltzmann’s constant (1.381 × 10^−23^ J/K), *h*: Planck’s constant (6.626 × 10^−34^ Js).

The application of Eyring’s absolute reaction rate theory model has not been widely used to describe frying process, but it is possible to describe some underlying mechanisms during this process. For example, Moyano and Zúñiga [99] used the enthalpy–entropy compensation approach based on Eyring’s absolute reaction rate theory to study the colour kinetics of French fries during frying. The results indicated that the activation entropy decreased during frying because of the limited space for molecular movement after drying.

## 6. Concluding Remarks and Future Directions

Frying is a multifaceted process involving the occurrence of heat, mass and momentum transfers that change the physical and chemical states of the food. The application of PEF treatment to plant-based foods is likely to enhance heat, mass and momentum transfers and is an effective emerging technology capable in controlling Maillard reaction and promoting starch gelatinisation, during frying. As a result, numerous quality improvements such as reduction in oil uptake and toxic compound in the final products and improvement in process efficiency such as decreases in energy cost and consumption, have been reported owing to the implementation of PEF technology in the frying industry. However, the underlying mechanisms of how PEF-treated plant materials withstand the frying process and its impact on quality parameters are not yet fully understood. Frying models are widely used to describe the frying process and to predict quality changes but frying models to describe the frying process of PEF-treated plant-based foods have yet to be developed. Building and validation of frying models, either observational, physical or based on kinetics, is of future research interest in order to explain better the underlying mechanisms and influence of PEF pretreatment on fried foods. Moreover, appropriate frying models can then be applied universally across the frying industry to aid the optimisation of PEF processing parameters on a wide range of plant materials in order to achieve the desired quality parameters in the final fried products.

## Figures and Tables

**Table 1:** Application of PEF on potato tissues followed by frying process.

Plant	Experimental Setup	PEF Parameters	Frying Parameters	Quality Parameters	Key Findings	References
Potato	PEF treatment was applied on peeled potatoes in cube form with dimension of 2 × 2 × 2 cm, followed by frying	*E* = 0.75, 2.50 kV/cm, *N* = 810, 9000 pulses, *W* = 18.9 kJ/kg, *t* = N/A, *f* = N/A, *τ* = N/A	Temperature = 190 °C, Frying time = 1 min	Weight, Moisture, Drip loss, Colour, Texture, and Oil content (fried)	Before frying: Moisture content=, Texture: peak force↓, Colour: lightness↓, a* ↑ After frying: Moisture content=, Texture: peak force=, Colour: lightness↑, a* ↓, Oil content ↓	[13]
Potato	Whole unpeeled potatoes were PEF-treated and then cut into 9 mm straight fries, followed by blanching, hot-air drying and finally par-frying	*E* = 1.0 kV/cm, *N* = N/A,*W* = 0.05, 0.2, 0.8, 1.0 kJ/kg, *t* = N/A, *f* = 1 Hz, *τ* = 75 μs	N/A	Cell disintegration index, Cutting force, Peeling loss, Feathering, Starch loss during cutting, and Fat uptake (par-frying)	Before frying:Cell disintegration index↑, Cutting force↓, Peeling loss= After cutting: Feathering↓, Starch loss↓ After frying: Fat content↓	[12]
Potato	Whole peeled potatoes were PEF-treated and then cut into 10 × 10 × 40 mm strips, followed by drying (100 °C for 10 min) and finally frying	*E* = 1.5, 2.5, 5 kV/cm, *N* = N/A, *W* = N/A, *t* = N/A, *f* = 2 Hz, *τ* = 100, 400 μs	Temperature = 190 °C Frying time = 3 min	Microscopic visualisation, Sugar content (sucrose, D-glucose, D- fructose) (before frying), Salt uptake (before frying), Drying efficiency (before frying), and Fat uptake (fried)	Before frying: Cell wall is affected, Sugar content↓, Conductivity and uptake of sodium chloride↑ After drying: Water content↓ After frying: Fat uptake↓	[30]
Potato	PEF treatment was applied on peeled potatoes in slice form with thickness of 1.5 mm, followed by frying	*E* = 1.5 kV/cm, *N* = 1000 pulses, *W* = N/A, *t* = 10 ms, *f* = 100 Hz, *τ* = 10 μs	Temperature = 175 °C Frying time = 3 min	Colour, Texture, and Acrylamide	After frying: Colour: hue angle↑, Texture: firmness↓, crispness↓, Acrylamide↓	[49]
Potato	PEF treatment was applied on potatoes in disk form with 25 mm diameter and 2.5 mm thickness, followed by vacuum drying (40–70 °C, up to 7200 s) and finally frying at low temperature for long time	*E* = 0.6 kV/cm, *N* = 10, 100 pulses, *W* = N/A, *t* = 10 ms, *f* = N/A, *τ* = 100 μs	Temperature = 130 °C * Frying time = Kinetic approach, up to 1400 s	Texture, Moisture content, and Oil uptake	After frying: Texture: firmness↑, peak force↑, Oil uptake↓	[14]
Potato	PEF treatment was applied on potatoes in disk form with and after cutting into disk form with 25 mm diameter and 2.5 mm thickness, followed by convective air drying (50 °C, up to 2000 s) and finally frying	*E* = 0.6 kV/cm, *N* = 10, 100 pulses, *W* = N/A, *t* = 10 ms, *f* = N/A, *τ* = 100 μs	Temperature = 130 °C * Frying time = Kinetic approach, up to 2000 s	Water content, Oil uptake, and Texture	After frying: Effective moisture diffusivity↑, Water content↓, Oil uptake↓, Texture: peak force↑	[59]

PEF parameters: *E* = electric field strength (kV/cm), *N* = number of pulses, *W* = specific energy input (kJ/kg), *t* = treatment time (ms), *f* = pulse frequency (Hz), *τ* = pulse width (μs). N/A = data not available. Key findings: ↑ increase, ↓ decrease, and = no change (all compared to their untreated counterparts). * Low frying temperature was intentionally used in these studies to investigate the quality of food material using a kinetic approach and is not recommended for actual practice.

**Table 2:** Summary of different types of mathematical models used in the literature to describe the frying process for solid plant foods.

Plant	Product Dimensions and Pretreatment	Frying Parameters	Model Type	Key Findings	References
Potato	Cylinder: 0.006 m diameter × 0.006 m length	*T* = 160, 190 and 220 °C; *t* = Kinetic approach, up to 240 s	A fractional conversion first-order kinetic and Arrhenius model	A two-stage rate process (the rapid and slow process) was found to describe the mechanisms of the water loss during frying.	[29]
Potato	Strips: 20 mm × 7 mm × 7 mm Preheating: 70 °C for 2 h	*T* = 135, 160, 175 and 190 °C; *t* = Kinetic approach, up to 12 min	An exponential decay and Arrhenius model	A high level of acrylamide was volatilised during frying, where acrylamide was detected in French fries, frying oil and air.	[87]
Potato	Slices: 2 mm thickness, 50 mm diameter	*T* = 170, 180 and 190 °C; *t* = 200–300 s; Location in the fryer: centre, 0.3 mm off-centre and 0.75 mm off-centre	A simple moving boundary model (Note: The core temperature was defined as a value of a region rather than a point)	The model was able to predict the temperature at different locations of the potato slice. The model considered the effect of varying convection heat transfer coefficients of oil.	[79]
Potato	2,3, 25.4 mm thick chip	*T* = 170, 180 °C; *t* = 50–800 s	A one-dimensional core-crust moving boundary model	The model was able to describe deep fat frying, air-drying, freeze drying, steam drying and spray drying.	[42]
Potato	2, 3, 4, 5, 6, 25.4 mm thick potato chips	*T* = 180 °C; *t* = 240 and 540 s	A one-dimensional moving boundary model	The frying process of multidimensional geometry for other food-types can be described with this model. The numerical finite difference method required the computation to start with the presence of two regions (crust and core). This was achieved by approximating the time required to form a thin layer of crust.	[78]
Potato	12 mm thick potato slices with an average diameter of 50 mm	*T* = 158 °C; *t* = 3300 s	A one-dimensional core-crust moving boundary model based on quasi-steady state analysis	The model showed the existence of a moving interface that recedes towards the core of the samples as time progresses during frying, freeze-drying and air-drying.	[88]
Potato	Uniform cylinders of three different diameters: 8.5, 10.5, and 14 mm	*T* = 140, 160, and 180 °C; *t* = 20, 40, 60, 120, and 180 s	A cylindrical crust-core frying model	A Nusselt relationship was applied for cylindrical bodies to describe the release of vapour bubbles during the frying process. The characteristic length and velocity for the Reynolds number were taken as the average diameter of the vapour bubbles and vapour bubble release frequency multiplied with the bubble diameter, respectively.	[80]
Potato	Slices: thickness 1.5 mm	Frying pressures and *T*: 1.33 kPa (118, 125, 140 °C), 9.89 kPa (118, 132, 144 °C), 16.7 kPa (118, 132, 144 ℃), and 101 kPa (150, 165, 180 °C)	A multiphase porous media model	Core pressure of potato slices reached approximately 40 kPa higher than the surface. Acrylamide formation was modelled on chip temperature rather than oil temperature.	[85]
Potato	Strips: 1.2 cm × 1.2 cm × 4 cm Coating with alginate gum: 0, 1, 1.5, and 2%	*T* = 170 °C; *t* = 590, 180, 270, and 360 s;	A 3D model	Effects of 0–2% alginate and four locations of potatoes on variables were studied. Moisture content and oil uptake in the coated samples were found to be significantly reduced.	[89]
Potato	Cylinders: 50 mm length, 8.5, 10.5, 14 mm diameter Soaked for 10 min in tap water	*T* = 140, 160, and 180 °C; *t* = 20, 40, 60, 120, and 180 s.	A pore inactivation model	The model was able to describe the overall trend of oil uptake compared to linear model. The model assumed that the moisture vapour from the porous crust inhibits oil migration.	[90]
Mushroom	Disk-shaped slices of 8 mm in thickness and 30 mm in diameter Osmotic dehydration (5 and 10%) and gum coating (2%)	*T* = 150, 170, and 180 °C; *t* = 0.5, 1, 2, 3, 4 min	A genetic algorithm–artificial neural network model (GA–ANN)	GA–ANN model was able to provide an accurate prediction for moisture and oil contents of fried mushroom.	[91]

Frying parameters: *T* = frying temperature; and *t* = frying time.

**Table 3:** Typical equations to model the frying processes.

Model Types	Mass Transfer	Heat Transfer	Momentum Transfer
Diffusion-based frying model	Fick’s law of diffusion	Arrhenius equation	N/A
Crust-core moving boundary model	1. Core region: Mass balance equation; Fick’s law of diffusion 2. Crust region: Mass balance equation; Idea gas law; Clausius–Clapeon equation	1. Initial heating period in both core and crust regions: Fourier’s equation 2. After crust formation: Core region: Fourier’s equation Crust region: modified Fourier’s equation including heat exchange and water vapour	N/A
Multiphase porous media model	1. Liquid: Mass balance equation; capillarity pressure (Kelvin’s law/ Flory–Rehner theory/Darcy’s law); gas pressure due to evaporation or gas release (Darcy’s law) 2. Gas (Vapour or air): Mass balance equation; molecular diffusion (Fick’s law); gas pressure (Darcy’s law) 3. Phase change of water: Hertz–Knudsen equation/Volume fraction continuity	Solid, liquid and gas: Energy balance equation (Fourier’s law of heat conduction)	Liquid and gas: Conservation of momentum; Darcy’s law (low permeability); Navier–Stokes equation; continuity equation

N/A = data not available.
